# Insights into the Management of Chronic Hepatitis in Children—From Oxidative Stress to Antioxidant Therapy

**DOI:** 10.3390/ijms25073908

**Published:** 2024-03-31

**Authors:** Ileana Ioniuc, Ancuta Lupu, Irina Tarnita, Alexandra Mastaleru, Laura Mihaela Trandafir, Vasile Valeriu Lupu, Iuliana Magdalena Starcea, Mirabela Alecsa, Ionela Daniela Morariu, Delia Lidia Salaru, Alice Azoicai

**Affiliations:** 1 Department of Mother and Child, “Grigore T. Popa” University of Medicine and Pharmacy, 700115 Iasi, Romania; ileanaioniuc@yahoo.com (I.I.); tarnita.irina@yahoo.com (I.T.); trandafirlaura@yahoo.com (L.M.T.); magdabirm@yahoo.com (I.M.S.); subotnicu_mirabela@yahoo.com (M.A.); agrudnicki@yahoo.com (A.A.); 2 Faculty of Medicine, “Grigore T. Popa” University of Medicine and Pharmacy, 700115 Iasi, Romania; alexandra.mastaleru@gmail.com (A.M.); deliasalaru@gmail.com (D.L.S.); 3 Faculty of Pharmacy, “Grigore T. Popa” University of Medicine and Pharmacy, 700115 Iasi, Romania; danamorariu2013@yahoo.ro

**Keywords:** children, chronic hepatitis, antioxidants, oxidative stress

## Abstract

Recent research has generated awareness of the existence of various pathophysiological pathways that contribute to the development of chronic diseases; thus, pro-oxidative factors have been accepted as significant contributors to the emergence of a wide range of diseases, from inflammatory to malignant. Redox homeostasis is especially crucial in liver pathology, as disturbances at this level have been linked to a variety of chronic diseases. Hepatitis is an umbrella term used to describe liver inflammation, which is the foundation of this disease regardless of its cause. Chronic hepatitis produces both oxidative stress generated by hepatocyte inflammation and viral inoculation. The majority of hepatitis in children is caused by a virus, and current studies reveal that 60–80% of cases become chronic, with many young patients still at risk of advancing liver damage. This review intends to emphasize the relevance of understanding these pathological redox pathways, as well as the need to update therapeutic strategies in chronic liver pathology, considering the beneficial effects of antioxidants.

## 1. Introduction

Since the evolution of photosynthesis, life has had to adapt to the environment’s oxidative reactions. Consequently, novel molecular mechanisms have been discovered to regulate and maintain the balance of reducing and oxidizing reactions. All these systems collectively constitute the phenomenon known as redox homeostasis. Redox interactions regulate a variety of biological processes, such as differentiation and development, metabolism, immunological responses, circadian rhythm, cell death, and others [1].

The liver is an essential organ in maintaining redox equilibrium. Several antioxidant reactions occur at the cellular and molecular levels. When pathological processes occur, this balance becomes disturbed and it favors pro-oxidative agents, which induce cell damage and changes that permeate into the nucleus, changing the cellular deoxyribonucleic acid (DNA). Various biochemical events occur in the liver, which is responsible for the majority of the human body’s metabolic activities. The results of these actions can disrupt the redox state and cause damage to the hepatocytes. Reactive oxygen species (ROS) can impact signal transduction and influence gene expression via antioxidant-responsive elements (AREs) [2].

Cells have stress response systems that help them maintain redox homeostasis under changing environments. Many of the downstream effector genes in these systems contain the antioxidant response element (ARE), which is recognized and controlled by the transcription factor Nrf2 [1]. ARE genes encode antioxidant enzymes, which include glutathione S-transferase (GST), nicotinamide adenine dinucleotide phosphate (NADPH), quinone oxidoreductase, and glutamate-cysteine ligase. Erythroid 2-related factor 2 (Nrf2) regulates the expression of ARE genes [3]. Nrf2 also regulates the expression of enzymes involved in redox homeostasis by upregulating exporters that may help eliminate electrophilic toxins from the cell, downregulating anabolic enzymes that compete with redox systems for NADPH, and modulating enzymes involved in controlling heme metabolism and iron homeostasis [1].

Nrf2 can protect the liver against damage that can cause acute and chronic hepatic illness, as well as aid in liver regeneration. Monitoring the ARE genes as biomarkers could help in the early diagnosis of liver illness. More research is needed in this area because each ARE gene may play an important role in maintaining redox homeostasis. Chronic liver diseases involve multiple hepatic cell types, including stellate cells, Kupffer cells, and sinusoidal endothelial cells [4,5].

It is widely recognized that the liver is involved in numerous metabolic activities via mitochondrial and microsomal systems. These typical metabolic activities result in the physiological production of reactive oxygen species (ROS) in hepatocytes [6]. As a defense mechanism, the organism utilizes antioxidant molecules such as catalase (CAT) and superoxide dismutase (SOD) [7], and lipid peroxidation causes an increase in malondialdehyde (MDA), a biomarker closely related to hepatocyte destruction [8]. Free radical species may be used as early markers of possible hepatotoxicity [9].

It has been recently discovered that vitamin D [10] and melatonin [11] have been shown to improve liver function in children with nonalcoholic fatty liver disease (NAFLD). Vitamin D has garnered specific attention as one of the contributing factors of autoimmunity because it acts as an immunomodulator and inhibits inflammation by reducing T and B cell auto-aggression. Lower vitamin D levels are common in several autoimmune diseases, including autoimmune hepatitis [12].

## 2. Viral Chronic Hepatitis and Oxidative Stress

Hepatitis C virus (HCV), a ribonucleic acid (RNA) virus, and hepatitis B virus (HBV), a DNA virus, are the leading causes of chronic liver injury. They can cause potentially deadly complications, such as cirrhosis and hepatocellular cancer [13]. Both viruses, HCV (Section 2.1) and HBV (Section 2.2), contribute to increased oxidative stress by causing chronic inflammation in the liver and activating immune cells and pro-inflammatory cytokines, all of which boost the formation of ROS.

Chronic HCV infections are typically associated with increased oxidative stress in liver tissue. An elevated level of oxidative stress may be a negative factor in liver injury and influence the severity of the disease [9,13]. Hepatocytes; nonparenchymal liver cells, notably Kupffer cells (resident macrophages); inflammatory cells; hepatic stellate cells (HSCs); and other immune effector cells are responsible for ROS production and liberation [14].

### 2.1. Hepatitis C Virus and Oxidative Stress

HCV, which belongs to the Flaviviridae family, is the etiologic agent of worldwide viral hepatitis, causing hepatocyte inflammation, fibrosis, cancer, and, ultimately, liver failure [15]. Since this condition is typically asymptomatic, enhanced screening procedures such as ELISA and the more accurate HCV-RNA PCR have been used for early diagnosis [16]. This virus can cause systemic oxidative stress and is responsible for ROS accumulation and oxidant–antioxidant imbalance. Two studies [17,18] have demonstrated that oxidative stress is higher in HCV than in other liver disorders and that antioxidants may be a therapeutic key to reducing the detrimental effects of hepatitis C. The HCV virus causes the generation of cytokines and tumor necrosis factor (TNF-α), leading to increased oxidative stress and ROS buildup. The primary ROS known to cause liver injury are O_2_ and H_2_O_2_ [19,20]. Nonstructural 3 protein (NS3), a hallmark protein of HCV, activates the mitochondrial electron transport chain via NADPH oxidase, which is responsible for T cell malfunction and damage to surrounding cells [21].

The highest infection rate was recorded in Egypt, where a cross-sectional study [22] was conducted. This study evaluated treatment-naive HCV-diagnosed patients and their changes in cellular hypoxia/angiogenesis, blood oxidative stress, and cellular immunological biomarkers versus healthy controls. Key factors of the immune system involved in oxidative stress response such as IgG and granulocyte/monocyte colony-stimulating factor (GM-CSF) were increased in the patients’ group, as well as angiogenesis/hypoxia factors such as vascular endothelial cell growth factor (VEGF) and lactate.

In infected hepatocytes, HCV increases ROS generation. The redox imbalance that occurs leads to DNA mutation and, finally, to cell death [23,24,25,26]. Physiopathological ways to lower HCV organism antioxidant defenses are through the activation of pro-oxidant enzymes, pro-inflammatory cytokines, and hepatic stellate cells. The next step in the cascade of biochemical changes at the cellular level is mitochondrial failure, a key point in the mechanisms that determine liver injury [27,28]. The HCV core protein is known to inhibit the mitochondrial electron transport chain and decrease intracellular and mitochondrial glutathione (GSH) levels, thereby causing oxidative stress [4,29,30]. The damage to the liver structure causes changes in blood flow, resulting in tissue hypoxia [31]. In the cascade of defense systems, the body activates hypoxia-induced vascular endothelial growth factors (VEGFs) and their counteracting soluble receptors. The hepatitis C virus can hamper VEGF activation through oxidative stress, which cause changes in VEGF mRNA expression. Under normal conditions, lactate is metabolized in the liver and kidney after it is produced in the peripheral tissues. If HCV infection occurs, it mainly influences the mitochondrial respiratory chain complex genes, which leads to the activation of anaerobic processes and lactate and ROS accumulation [4,32]. This virus causes the destruction of the hepatocyte through multiple mechanisms, mainly by counteracting the activity of cellular defense factors. It is responsible for immunological abnormal responses such as cytokine changes, autoantibodies, and hypergammaglobulinemia.

It was demonstrated that HCV infection does not only promote the oxidative stress, but the virus replication is also closely related to the redox changes that occur in the cell [33]. Some studies argued that HCV induction of oxidative stress can be related to the incidence, severity, and persistence of the infection [24,25,26,27]. 

Regarding the level of enzymes involved in oxidative stress, Mohamed Ahmed Khedr et al. [29] monitored the serum levels of glutathione peroxidase and malondialdehyde, both being low in pediatric patients with chronic hepatitis C (CHC). Another study [30] showed high levels of oxidative stress markers and ferritin and low levels of vitamin D in adults with CHC.

In hepatitis C virus-infected adolescents with beta thalassemia major, Mona S Abdel et al. [32] reported a significant decrease in aspartate aminotransferase (AST), as well as an improvement in total antioxidant capacity and serum levels of malondialdehyde in the group treated with metformin, but without significant clinical improvement in the patients’ condition.

### 2.2. Hepatitis B Virus and Oxidative Stress

Despite the medical world’s efforts to prevent and treat hepatitis B, we continue to be far from eradicating the illness. We are unable to manage this disease unless we completely grasp all of the mechanisms involved. Oxidative stress is a critical component associated with the numerous pathogenic processes in HBV infection [34].

The Food and Drug Administration (FDA) had approved only five medications for treating hepatitis B in children in the United States by 2020: interferon alpha, tenofovir disoproxil, lamivudine, entecavir, and peginterferon alfa-2a [35]. Since 2022, the FDA has approved alafenamide as a tenofovir supplement for children aged 12 and higher [36]. These medications primarily inhibit viral multiplication and are partially effective in slowing disease progression to hepatocellular carcinoma (HCC) and cirrhosis [37]. Under these conditions, it is critical to emphasize the importance of new drugs and treatment practices.

Oxidative stress a state that causes much cell destruction, affecting lipids, proteins, and DNA, and it is considered the main cause of many diseases and cancers [4]. Because of their double bonds, lipids are the most vulnerable to ROS attack. Their end products could damage the cell membrane, DNA, and proteins. The most studied lipid peroxidation product is MDA, and it is used as an oxidative stress indicator [29]. The MDA level was found higher in HBV infection compared with healthy subjects and it was positively correlated with alanine aminotransferase (ALT) [38,39], total and direct bilirubin [40], gamma-glutamyl transpeptidase (GGT) [38], serum iron/ferritin [41], HBV-DNA [34,42], and liver ultrasonography findings (coarse texture) [34] in some studies.

Severi et al. [43] found that even though HBV virus produced significant oxidative stress, lipid peroxidation product levels were not modified. This finding intrigued the authors to look for these peroxidation products and their effects outside the liver. Another study [44] reported an increased prevalence of carotid atherosclerosis in patients with HBV. In all the studies mentioned, a significant rise in erythrocyte or plasma MDA levels was found, but none of them measured the MDA levels in hepatic tissue. There is little evidence [45] of lower MDA levels in HCC patients caused by HBV infection.

ROS can also cause protein lesions, and the ones most used as markers for oxidative stress end products are carbonylated proteins (protein CO) [46]. Studies in the protein oxidation area are rare. More findings in this field could help us to understand the source and severity of oxidative stress processes. As an example, the presence of NO and superoxide [45] can be demonstrated by nitrotyrosyl residue measurement. There are controversial results between two studies [37,41] regarding the nitrotyrosyl levels in HBV patients. The first one did not report any difference between healthy subjects and HBV patients and the second one demonstrated a significant increase in the serum nitrotyrosyl levels in chronic HBV. These findings indicate an increased amount of nitric oxide in the hepatic tissue of the studied patients.

Although the carbonyls can be produced by almost all ROS and the source of oxidative stress cannot be identified, some studies showed that their levels in chronic active and inactive hepatitis B patients were increased [42]. Moreover, Popadiuk et al. [47] revealed significantly enhanced plasma carbonyl levels in pediatric patients with chronic hepatitis B infection.

The DNA damage produced by ROS leads to genome instability and then to mutations [48]. Shimoda R. et al. [49] found increased levels of 8-hydroxydeoxyguanosine (8OHdG) in human livers with chronic hepatitis B (CHB) and also showed a positive correlation between ALT levels and the mutations’ contents. In disease progression from the F1 stage to F4 stage, increased oxidative DNA damage was found, leading, eventually, to HCC occurrence. DNA lesions were also identified in the peripheral blood lymphocytes in both HCC and CHB. 

In a large study [50] made in Han, China, six genetical variants linked to oxidative stress (CYBA-rs4673, NCF4-rs1883112, NOX4-rs1836882, rs3017887, SOD2-rs4880, and GCLM-rs41303970) were found and their effect on HBV liver disease was studied. The subjects were divided into five groups: healthy controls, chronic hepatitis B (CHB), liver cirrhosis (LC), hepatocellular carcinoma (HCC), and natural clearance. Gene–gene interactions were found between healthy controls and the CIB group (CHB+LC+HCC). Gene polymorphism, which is caused by oxidative stress, is highly associated with HBV-induced liver disease [50].

### 2.3. Chronic Hepatitis C Virus vs. Chronic Hepatitis B Virus

One of the features that differentiates HCV from HBV is its potential to induce oxidative stress in the infected cells. Moreover, HCV reduces the expression of the hepcidin gene, which is responsible for slowing down the iron inflow from the gastrointestinal tract. These changes lead to liver iron overload and to the continuous propagation of oxidative stress. ROS cause changes in protein architecture, making them more susceptible to oxidative attack, because of the formation of carboxyl groups [50,51].

A previous study [52] analyzed the oxidized modifications of proteins (OMPs) in 94 children diagnosed with CHC and 9 children with cirrhosis of HCV etiology. The attention of this study was focused on the formation of ketonic and aldehyde groups after protein interaction with 2.4 dinitrophenylhydrazone (2.4 DNPH). The main purpose of the study was to examine which protein oxidation takes place in healthy children and which takes place in ill children. The level of ketondinitrophenylhydrazones (mKDNPH) and aldehydedinitrophenylhydrazones (mADNPH) was found to be significant low in both groups. The level of OMP products was doubled in children with CHC and increased by 2.5 times in those with cirrhosis. The main changes observed in ill children was in the levels of mKDNPH (a biomarker that shows the degree of oxidative protein destruction), which were very high: in CHC patients it increased by 150% and in cirrhosis patients by 200%, while in healthy subjects the level was at 28% [52]. 

Although CHC in children induces oxidative stress, proved by OMP activation, the intensity of this process was observed to be higher in patients with a longer period of illness and active CHC. These evaluation criteria could be used for the evaluation of the pathological progress of liver disease [29].

It has been shown that on lymphoid cells, ROS can cause disruption of antiviral response and therapy resistance, favoring chronic infection [53]. 

In hepatitis B, the increase in oxidative stress is caused mainly by pro-inflammatory cytokines. These patients have high levels of TNFα, which is correlated with increased superoxide production in mitochondria. CHB subjects also have low glutathione (GST) activity, which is proportionally related to the progression of hepatocellular carcinoma (HCC) [54]. Moreover, oxidative stress can affect viral DNA, leading to the earlier development of HCC [55,56]. 

In order to slow down oxidative processes by using antioxidant assays—among which were glutathione (GSH), superoxide dismutase (SOD), thiobarbituric acid (TBA), superoxide anion radical scavenging activity, radical cation assay, and 2,2-diphenyl-1-picrylhydrazyl (DPPH) radical cation assay—a study [57] was conducted. These antioxidant levels were found lower than the control group, while MDA levels were higher in ill patients. 

Pinar Cıragil et al. [22] spectrophotometrically measured the levels of CAT, SOD, and MDA to evaluate the oxidant/antioxidant activity in serum of 20 patients diagnosed with CHB and CHC and 30 patients from the control group. The results showed that in patients with CHC, the levels of SOD and CAT were lower than those of the patients with CHB. The levels of these enzymes were higher compared to the control group. Regarding MDA levels, these were more increased in CHC patients compared to CHB patients. The results of this study once again validate previously conducted studies [58,59,60], which showed that mononuclear cells from peripheral blood had increased MDA levels and low SOD activity in subjects with CHC. Increased MDA levels were also found in CHC patients before interferon treatment compared with healthy subjects. However, the CAT and SOD activity was lower in patients with CHC than in those diagnosed with CHB [22]. 

Although MDA levels were also higher, the CAT and SOD activity was lower than the control group in CHB subjects. These findings highlight the importance of redox homeostasis. When organism defense mechanisms are overcome, ROS have harmful effects and, as a consequence, hepatocyte lesions occur. An inadequate antioxidant barrier enhances the oxidative stress in CHC and CHB patients [22]. These studies were further supported by several authors [61,62,63], the results being summarized below in Table 1. 

In Cengiz et al.’s study [55], the total antioxidant response (TAR) of HBV patients with or without cirrhosis was evaluated. The results showed that TAR was low in patients with cirrhosis compared with the control group. Regarding ALT values and their correlation with TAR, the authors could not find any connection.

p38 mitogen-activated protein kinase (p38-MAPK) and nuclear factor kappa-light-chain-enhancer of activated B cell (NFkB) pathways can be changed by pre-S mutation, which, in turn, influences COX-2 activity. High COX-2 level was found to be responsible for many types of malignancies and for disease progression [64]. 

Hui-Ching Wang et al. [56] performed a cohort study on HBV patients. The main purpose was to evaluate the effect of pre-S mutants and oxidative stress in ER and their role in the progression of chronic HBV infection, cirrhosis, and HCC. The authors concluded that Pre-S mutants induce endoplasmic reticulum (ER) oxidative stress, leading to cell destruction, fibrosis, and progression to HCC.

Lately, the implication of oxidative stress in disease progression has been heavily studied and included in the eligibility criteria for antiviral treatment. ROS lesions in hepatocytes occur through many biochemical processes, such as increased mitochondrial ROS production/SRN by the electron transport chain bound to nonstructural 5A proteins (NS5A) and cores; reduced production of GSH due to liver damage; activation of NAD(P)H oxidase in Kupffer cells and polymorphonuclear cells during inflammation; activation of NAD(P)H oxidase by the NS3 protein; iron overload and lipid peroxidation; increased expression/activity of Cox-2; decreased number of antioxidants in cells and reduction in their gene expression; increasing cytokine and SRO augmentation; increased CYP2E1 expression; and alcohol, drugs, other toxic elements [65].

Lipid peroxidation compounds are the main ROS that have been studied, and some authors [55,66] examined their blood and urinary levels together with vitamins C, A, E, and selenium in hepatitis C infection. These compounds were found at very high levels, while glutathione was significantly low in mitochondria. Antioxidants levels deceased proportionally to age in the control group. The fibroscan score was positively correlated with urinary values of 8-isoprostane, and negatively with the level of vitamin A, implying that antioxidant therapy could slow down the progression of CHC.

The core HCV gene was found to directly impact mitochondrial cytochrome C, causing ROS redistribution in cytosol and damage at this level [67]. Alpha-GST blood levels can be used to assess active hepatic injury because its level increases in active injury and decreases rapidly when the aggression stops [68].

Pediatric population studies [39,65] found a more significant increase in the oxidative stress parameter values of CHC patients rather than CHB patients. Although antiviral therapy (interferon (IFN) and ribavirin) does not have direct antioxidant proprieties, it can slow down the ROS production by its anti-inflammatory action [55,67].

In order to establish the antioxidant therapy efficiency in HCV patients, the plasma antioxidant status was assessed by Mirelle S. et al. [69] before and after the supplementation with zinc and vitamin C and E. The patients were divided into three groups: control group, patients without antiviral treatment but with daily supplementation with antioxidants (vitamin E 800 mg, vitamin C 500 mg, zinc 40 mg) for 6 months, and patients treated with pegylated interferon and ribavirin. Before the IFN and ribavirin treatment, subjects had high levels of SOD, CAT, and glutathione peroxidase (GPX). The activity of glutathione reductase was lower and the products of lipid peroxidation were higher than in the control group. After antioxidant supplementation, CAT and glutathione S-transferase activities, together with lipid peroxidation, were lower. Before antioxidant therapy, transaminases and GGT showed a significant increase. These findings concluded that antioxidant therapy could play a key role in the management of both types of viral hepatitis (HCV, HBV). 

## 3. Other Chronic Hepatitis

### 3.1. Wilson Disease (WD)

Wilson disease (WD) is an autosomal recessive pathology that mainly affects the liver. The ATP7B gene is responsible for this disease and it encodes the copper transport via P-type ATPase. This transport is then affected, and together with the low levels of ceruloplasmin, copper accumulation in the liver occurs [70]. As a result of liver loading with copper, more free radicals are produced and the oxidative stress causes hepatocyte lesions and progression to liver failure [71,72].

Nagasaka H. et al. [71] conducted a study on 13 pediatric patients with WD presenting with hepatic dysfunction to investigate the liver’s antioxidant responses in WD. Decreased levels of oxidized glutathione (GSSG) and reduced glutathione (GSH) and increased levels of lipid peroxidation products and thiobarbituric acid-reactive substance (TBARS) were found in all patients. Antioxidant enzymes such as Mn-SOD, CuZn-SOD, and CAT were also decreased. Asymptomatic carriers had normal levels of GHS, GSSG, and thiobarbituric acid-reactive substances (TBARS) and increased levels of antioxidant enzymes. All together, these results underline that copper-related oxidative stress is an important factor in liver disease progression in WD patients.

### 3.2. Autoimmune Hepatitis

Autoimmune hepatitis (HAI) is caused by an immune system dysfunction that leads to abnormal antibody production against hepatocytes. This pathology is more frequent in females [73]. The liver inflammation that occurs—as well as the disease’s progression to its final stages: cirrhosis and hepatic failure (if the treatment is delayed)—can be histopathologically evaluated [74]. The treatment of this pathology is based on immunosuppressants, which have proven their effects in clinical symptom amelioration and even in hepatic fibrosis reversion [75]. Studies have shown that there is a tight bond between inflammatory response and oxidative stress in autoimmune hepatitis patients [74,76]. Vitamin D is an important antioxidant, and its deficiency seems to be a key factor in the occurrence and development of autoimmune hepatitis. A study [76] showed lower levels of SOD, total antioxidant capacity (TAC), and T cells, while the levels of total bilirubin, its fractions, and MDA were higher in the observed group than controls. The conclusion was that patients with decreased levels of vitamin D are more susceptible to oxidative stress because of the decreased levels of T cell subsets and immune system response.

## 4. Treatments and Antioxidants Used in Chronic Viral Hepatitis

Many drugs, such as antidepressants, anti-inflammatory, anti-analgesic, and anti-cancer drugs have increased cellular oxidants and decreased cellular antioxidants in the liver of experimental models [9]. The majority of drugs are metabolized in the liver, making it more susceptible to damage [77]. For example, paracetamol can determine an MDA level increase in the liver and a remarkable reduction in SOD activity. Gallic acid has been proven to have antioxidant and hepatoprotective effects in mice with liver damage caused by paracetamol [78]. 

The most studied vitamin with antioxidant effect is vitamin E, followed by nutritional antioxidants such as zinc and coenzyme Q10. N-Acetyl-L-Cysteine (NAC), silymarin, metadoxine, and mitoquinone were used as drug supplements for liver pathologies. Some plants and foods can also have an antioxidant effect in liver disease: chocolate, ginger, and green tea [9,79]. Two well-studied [80,81] natural bioactive components, quercetin and resveratrol, have been studied as a food supplement for patients with liver pathologies. More studies need to be conducted in this therapeutic area.

It has been demonstrated that selenium has more antioxidant activity than vitamin E. Selenium can act on hydrogen peroxide and free radicals and repair damaged sites [79]. Moreover, vitamin C can reassemble the reduced form of vitamin E [78]. 

Liver damage caused by D-galactosamine can be slowed down in experimental models by using an antioxidant combination of alpha-tocopherol, selenium, ascorbic acid, betacarotene, and ubiquinone [82,83]. Some antioxidants such as linoleic acid, vitamin D2, and beta-carotene are considered potential adjuvants for interferon therapy because they can reduce HCV activity in mice hepatocytes [84].

Some new therapeutic strategies propose the use of antioxidant enzymes including GSH, SOD, and CAT; vitamins A, C, and E; chemical antioxidants such as tannins, coumarins, and alkaloids; flavonoids; and phenolic compounds because of their properties that sustain cells’ antioxidant defense system [85,86,87,88,89,90,91,92,93,94,95,96,97].

In CHC patients, silymarin alone was documented to reduce oxidative stress, as well as in combination with another two potent antioxidants (selenium and alpha-lipoic acid) [88,89,90]. These results were sustained by another study [91] conducted on 170 patients who took 420 mg/day of silymarin for 41 months. In these patients, a significant improvement in cell survival was documented [91,92]. Silymarin also has antiviral action, reducing hepatitis C virus replication [93].

Resveratrol was found not to be effective as antioxidant treatment for CHC because it can extend HCV RNA replication [94]. In a tissue culture, quercetin was studied for its capacity to reduce the production of viral proteins, slowing down the spread of the virus. A more recent study [95] on six mice models with induced liver fibrosis used intraperitoneal injections with mitoquinone (mitochondrial-targeted antioxidant) to evaluate its capacity to inhibit ROS. The paper concluded that mitoQ markedly reduced the expression of profibrogenic transforming growth factor-β as well as type I collagen, leading to the attenuation of liver fibrosis by inhibiting ROS production and the JNK/YAP signaling pathway. 

Interferon therapy is effective in HBV viraemia suppression, but a high probability of reactivation after the end of treatment was documented. Both viruses determine ROS generation and oxidative stress in infected cells—which has been extensively studied in many papers [55,95,96,97]—leading to liver failure. Moreover, oxidative stress disturbs the immune response of the host against pathogens [98].

Vitamins have been documented as powerful antioxidants, this role being able to be used in the exploitation of future therapeutic strategies [99,100,101]. They are also responsible for regulation of both adaptative and innate immune response through multiple pathways. The main process in which they take part is T lymphocyte differentiation into lymphocyte T helper 1 and 2 (Th1 and Th2), leading to cytokine activation and lymphocyte proliferation. Serum vitamin D, C, E, or beta-carotene deficit has been documented in viral hepatitis [102,103,104,105]. As mentioned above, in chronic hepatitis, a liver iron and serum overload occur, which is responsible for disease progression or lack of IFN therapy response. 

Vitamin B1 (thiamine) was documented for the formation of the dihydrolipoate complex, which is able to remove ferritin-bound iron [106,107]. Vitamin B deficiency is very common in cirrhosis [108]. Also, thiamine is believed to slow down the progression of hepatitis B. Two studies [109,110] were conducted in Chinese children to show the vitamin B efficiency in slowing down pathogenic progress or viral HBV seroconversion. In the first study [109], two groups of HBe-positive children were examined: those with IFNa-2a treatment and those with placebo thiamine syrup for 12 weeks. The results showed the same rate of viral DNA or HBe Ag loss. The second study [110], conducted on 19 HBe-positive children, was also divided into two groups: IFNa-2a with or without prednisone treatment or vitamin B complex as a placebo. In the study, no seroconversion was found in the placebo group. The controversial results lead to the conclusion that no benefit is shown from thiamine supplementation in pediatric chronic hepatitis B patients. In another study conducted on three adults [111], thiamine administration determined the normalization of the transaminases, but these are insufficient data to be able to conclude on the effectiveness of the use of vitamin B. 

Vitamin E is a liposoluble vitamin, and its main characteristic is that it has antioxidant activity by neutralizing ROS and free radicals [112,113,114]. It also has an important role in immunomodulation, influencing cell-mediated immunity. Andreone P. et al. [115] analyzed 32 adults who were HBe Ag-negative, positive, or non-responsive to the previous IFN treatment. The patients received vitamin E or no treatment and, at the end, seven patients from the vitamin E group had ALT normalization and undetectable HBV-DNA. Another study [116] showed that 23 pediatric patients out of 122 obtained HBe Ag seroconversion after vitamin E treatment. These results show that vitamin E could favor HBe Ag seroconversion, but larger studies are needed to emphasize the usefulness of vitamin E supplementation in the treatment of children with hepatitis B. 

Table 2 summarizes the main effects of antioxidants used as adjuvant therapy in chronic hepatitis.

Several studies [117,118,119,120] have investigated the efficacy of vitamin E in CHC patients. Alexandra von Herbay et al. [117] administered vitamin E to 23 patients who were non-responders to the previous IFN treatment. At the end, 11 patients (48%) were considered responders because the level of serum ALT was lower. None of the patients had ALT normalization or RNA-HCV negativity [117]. Another study [118] conducted on 24 patients who received IFNa-2a; IFNa plus N-acetylcysteine (NAC) and sodium selenite; or IFNa plus NAC, sodium selenite, and vitamin E. In the study, a complete response (ALT normalization and RNA-HCV negativity) was observed in 37.5% of patients receiving IFN as monotherapy; in 25% of patients treated with IFN plus NAC and sodium selenite; and in 75% of patients treated with IFN plus NAC, sodium selenite, and vitamin E. The association between IFN and vitamin E was found not to be effective because the virological and/or biochemical relapse occurred 6 months after the end of the treatment [118]. Ideo G et al. [121] concluded that vitamin E does not have an important role in preventing hepatic fibrosis. In the article, no difference between the studied groups was observed. The study included 120 CHC non-responder patients who were previously treated with IFN. They were randomized to receive IFN with or without NAC and vitamin E for 6 months. Recently, in order to evaluate the liver anti-inflammatory effect of vitamin E, a study conducted by Katia Falasca et al. [119] analyzed naive CHC patients who were divided into two groups: in one group, patients received no treatment, and in the second group, they received silybin-phospholipids and vitamin E complex for 3 months. In the treated group, a significant increase in serum IL-2 and decreasing in IL-6 was observed, which lead to a preferential shift to Th1 defense mechanisms. The conclusion of the authors was that the silybin-phospholipids and vitamin E complex exert liver anti-inflammatory effects [119].

The effectiveness of all-trans retinoic acid (ATRA) is has been measured in studies conducted on HCV replicon [122,123]. One of these studies [123] showed a downregulation of the replicon. The patients were randomly examined from a group with HCV genotype 1 diagnosis, which has low/non-response to previous antiviral treatments. They received combination therapy with either ATRA plus sodium selenite or a combination of ATRA, sodium selenite, and pegylated interferon alfa-2a (PEG-IFNa-2a). At the end of the treatment, the virological response, defined by RNA-HCV negativity, was obtained only in one patient in the ATRA group versus four patients in the ATRA plus PEG-IFNa group. Based on these results, ATRA could be a potential additive or synergistic therapy with PEGIFNa. Not all antioxidants have a beneficial outcome in hepatitis B therapy. Used as an adjuvant treatment, N-acetylcysteine (NAC) was found to be safe and efficient in HBV-diagnosed patients [124,125]. 

Fan et al. [126] demonstrated a major decrease in MDA levels in HB patients treated with interferon alpha-2b. In the same way, a study conducted on CHC patients wanted to determine the effect of pegylated interferon alfa-2b plus ribavirin combination therapy on oxidative stress [41]. The lipid peroxidation products could be decreased, and the antioxidant enzymes increased by the combination of interferon alpha and lamivudine [119]. Another study [61] conducted on patients who were receiving interferon alpha showed an increase in beta-carotene and glutathione levels together with a decrease in MDA levels during treatment. A complete resolution of oxidative stress was reported six months after INF-α therapy in this study. An increased level of antioxidant enzymes after antiviral treatment has been documented by some researchers [84].

There is a fine balance between good and harm when it comes the subject of the utility and dosage of antioxidants. For example, high doses of vitamin C can have pro-oxidant activity [127]. Another study [128] showed an increased incidence of lung cancer in smokers that used retinol plus beta-carotene therapy. There is a study [115] which showed that hepatitis B patients mostly have a low dose of antioxidants before therapy initiation. There was also evidence of one key viral protein, hepatitis B virus X protein (HBx), that can induce the activation of the transcription factors leading to the gene expression in the nucleus [129].

Alavian et al. [114] elaborated an antioxidant therapeutic strategy called “2-step Combined Antioxidant Adjuvant Therapy for hepatitis B”. Step one is considered the initiation phase, which focuses on strengthening and compensating the antioxidant system deficiencies. Step two is called the “maintenance phase”, which focuses on ongoing oxidative stress that occurs throughout the disease’s progression. This step has the aim of counteracting further oxidative damage. 

## 5. Conclusions

The liver’s unique anatomical location and functions make it vulnerable to various types of chronic injury from childhood. Because of its intracellular antioxidant system, it has an exceptional ability to neutralize free radicals produced during the metabolism of a wide range of medications and toxins. Chronic liver damage in children can be caused by a variety of conditions, the most common of which are discussed in this review: viral hepatitis C and B, Wilson disease, and autoimmune hepatitis. In chronic viral hepatitis, oxidative stress is increased not only by the virus itself, which has the ability to modify the genome of the hepatocyte to produce more ROS, but also by the inflammatory processes that result from the infection of the hepatocytes.

Increased free radical production and decreased antioxidant defense in hepatocytes accelerate the development of oxidative stress, which causes a range of liver dysfunctions including cirrhosis, necrosis, fibrosis, viral hepatitis, and hepatocellular cancer. The mechanisms that cause the appearance of a redox imbalance in chronic liver diseases are numerous and complex, and understanding and studying them in depth is essential, given the encouraging results of recent studies on the supplementation of classical therapeutic schemes with various antioxidants. Despite the origin of chronic liver disease, the formation of ROS and other free radicals is an essential component of oxidative stress. As a result, it remains challenging to avoid the generation of free radicals and oxidative stress in patients with chronic liver disease, as new therapeutic techniques and further research are required before specific protocols can be established.

## Figures and Tables

**Table 1:** Antioxidant enzymes levels in chronic hepatitis vs. MDA levels.

Authors	Patients	MDA Levels	GSH Levels	SOD Levels	CAT Levels
N Aslam et al. [57]	36 patients: 12 patients with CHC 12 patients with liver cirrhosis 12 controls	↑	↓	↓	-
P Cıragil et al. [22]	70 patients: 20 diagnosed with CHC 20 diagnosed with CHB 30 controls	↑	-	↓↓ CHC ↓ CHB	↓
I Dikici et al. [61]	71 patients: 23 acute HCV infection 23 controls 25 CHC	↑	↓	-	-
G Levent et al. [62]	47 patients: 19 CHC 28 controls	↑	↓	↓	-
AM Chrobot et al. [63]	100 patients with CHC	↑	↓	↓	↓

**Table 2:** Antioxidants used as adjuvant therapy and their effects.

Antioxidants Used as Adjuvant Treatment in Chronic Hepatitis:	Effects:
Vitamin E (alpha-tocopherol)	Together with alpha-tocopherol, selenium, ascorbic acid, beta carotene, and ubiquinone, slowed down the liver damage caused by D-galactosamine [82,83]. Sustains the cell antioxidant defense system [112,113,114,115]. Has antioxidant activity, by neutralizing ROS and free radicals [112,113]. Has an important role in immunomodulation, influencing cell-mediated immunity [115]. Could contribute to ALT normalization and to decreasing of HBV-DNA viremia [115].
Vitamin A, vitamin C, flavonoids, and phenolic compounds	Sustains the cell antioxidant defense system [85,86,87].
Selenium	Can act on hydrogen peroxide free radicals and repair damaged sites [79].
Vitamin D2	Potential adjuvant for interferon therapy because it can reduce HCV activity in hepatocytes [84].
Beta-carotene	Potential adjuvant for interferon therapy because it can reduce HCV activity in hepatocytes [84].
Silymarin	Reduces oxidative stress alone [88,89,90] Significantly improved the cell survival [91,92]. Has antiviral action, reducing hepatitis C virus replication [93].
Quercetin	It has the capacity to reduce viral extension and viral protein production in a tissue culture [94].

## Data Availability

No new data were created.
